# Antioxidant and Anti-Inflammatory Properties of Cherry Extract: Nanosystems-Based Strategies to Improve Endothelial Function and Intestinal Absorption

**DOI:** 10.3390/foods9020207

**Published:** 2020-02-17

**Authors:** Denise Beconcini, Francesca Felice, Angela Fabiano, Bruno Sarmento, Ylenia Zambito, Rossella Di Stefano

**Affiliations:** 1 Department of Life Sciences, University of Siena, via Aldo Moro 2, 53100 Siena, Italy; 2 Cardiovascular Research Laboratory, Department of Surgery, Medical, Molecular, and Critical Area Pathology, University of Pisa, via Paradisa 2, 56100 Pisa, Italy; francesca.felice@for.unipi.it; 3 Department of Pharmacy, University of Pisa, via Bonanno 33, 56100 Pisa, Italy; angela.fabiano@unipi.it (A.F.); ylenia.zambito@unipi.it (Y.Z.); 4 i3S-Instituto de Investigação e Inovação em Saúde, University of Porto, Rua Alfredo Allen 208, 4200-153 Porto, Portugal; bruno.sarmento@ineb.up.pt; 5 INEB—Instituto de Engenharia Biomédica, Universidade do Porto, Rua Alfredo Allen, 208, 4200-135 Porto, Portugal; 6 CESPU, Instituto de Investigação e Formação Avançada em Ciências e Tecnologias da Saúde, Rua Central de Gandra, 1317, 4585-116 Gandra, Portugal; 7 Interdepartmental Research Center Nutraceuticals and Food for Health, University of Pisa, via Borghetto 80, 56100 Pisa, Italy

**Keywords:** cherry, nutraceuticals, polyphenols, antioxidant, anti-inflammatory, intestinal absorption, nanoparticles, nanosystems, HUVEC

## Abstract

Cherry fruit has a high content in flavonoids. These are important diet components protecting against oxidative stress, inflammation, and endothelial dysfunction, which are all involved in the pathogenesis of atherosclerosis, which is the major cause of cardiovascular diseases (CVD). Since the seasonal availability of fresh fruit is limited, research has been focused on cherry extract (CE), which also possesses a high nutraceutical potential. Many clinical studies have demonstrated the nutraceutical efficacy of fresh cherries, but only a few studies on CE antioxidant and anti-inflammatory activities have been carried out. Here, the results concerning the antioxidant and anti-inflammatory activities of CE are reviewed. These were obtained by an in vitro model based on Human Umbilical Vein Endothelial Cells (HUVEC). To clarify the CE mechanism of action, cells were stressed to induce inflammation and endothelial dysfunction. Considering that antioxidants’ polyphenol compounds are easily degraded in the gastrointestinal tract, recent strategies to reduce the degradation and improve the bioavailability of CE are also presented and discussed. In particular, we report on results obtained with nanoparticles (NP) based on chitosan derivatives (Ch-der), which improved the mucoadhesive properties of the chitosan polymers, as well as their positive charge, to favor high cellular interaction and polyphenols intestinal absorption, compared with a non-mucoadhesive negative surface charged poly(lactic-co-glycolic) acid NP. The advantages and safety of different nanosystems loaded with natural CE or other nutraceuticals are also discussed.

## 1. Introduction

**Figure 1:**
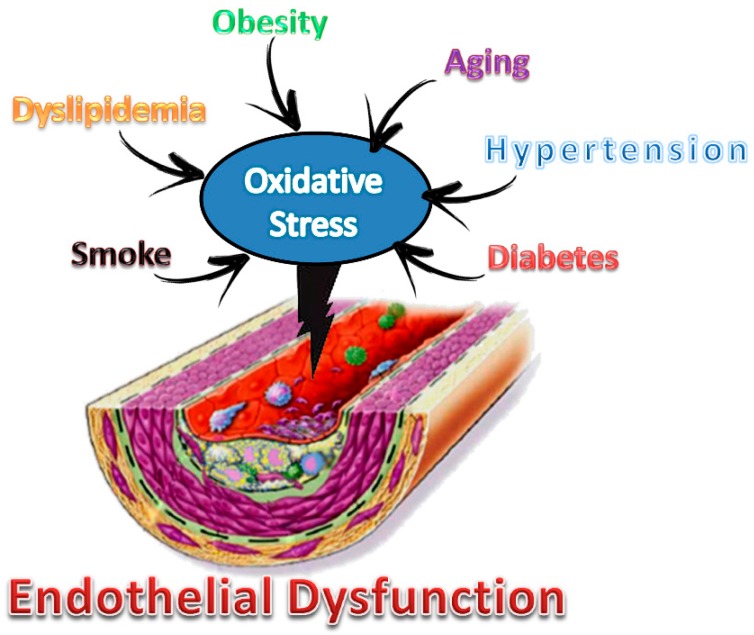
Main cardiovascular risk factors and their involvement in endothelial dysfunction.

Cardiovascular diseases (CVD) have always been recognized as the leading cause of death and invalidity in the Occidental world. Atherosclerosis (ATS), a fibroproliferative inflammatory disease due to endothelial dysfunction, is considered the major cause of CVD. Cardiovascular risk factors such as smoking, hypertension, dyslipidemia, diabetes, obesity, and a sedentary lifestyle lead to oxidative stress, which is the most important known factor involved in endothelial dysfunction (Figure 1).

Recent studies have demonstrated that a Mediterranean-type diet has a preventive effect on ATS and CVD [1,2,3]. In particular, the consumption of nutraceuticals contained in plant derivatives has showed a very important role in preventing ATS plaque formation. Among agri-food products, soft fruit such as strawberries, grapes, apples, cherries, etc. are widely consumed due to their good taste based on the balance between sugar and acid content in the fruit. Among soft fruit, sweet cherries (*Prunus avium* L.) have been studied for their high content in biologically active substances, such as phenolic acids. It is known that p-coumaric, p-hydroxybenzoic, chlorogenic, ferulic, and gallic acid, which are found in a lot of different sweet cherry cultivars, have antioxidant properties. Indeed, antioxidants have strong scavenging activity for superoxide and 2,2-diphenyl-1-picrylhydrazil (DPPH) radicals. Moreover, sweet cherries have an anti-inflammatory effect principally due to a decrease in plasma C-reactive protein (CPR) and nitric oxide (NO) levels [4].

However, a low bioavailability is the major problem of using antioxidants from cherry extract in therapy. A poor intestinal absorption along with oxidation in the gastrointestinal tract (GI) and marked metabolism in liver make it unlikely that high concentrations of these antioxidants are found in the organism for long after ingestion and reach the blood, which is the action site. From here, the notion came of preparing nanoparticles loaded with these natural extracts. This nanosystem prolongs the polyphenols residence in the GI lumen, reducing the intestinal clearance mechanisms and increasing the interaction with the intestinal epithelium, which is the absorption surface. Moreover, the nanoparticles can penetrate the tissues through the capillaries and are internalized in cells [5]. 

Despite the enormous success and consequent use of many synthetic polymers to prepare nanoparticles, using this polymer type in the nutraceutical field is not advisable, as substances of natural origin are required for this purpose. For this reason, we will only review nanosystems that are based on polymers of natural origin (chitosan and its derivatives), made of endogenous monomers (poly(lactic-co-glycolic acid)), or consist of natural phospholipids (liposomes).

## 2. Cardiovascular Diseases 

CVD are disorders that include coronary heart disease, cerebrovascular disease, and peripheral vessel disease. According to the World Health Organization (WHO) report [6], CVD have been responsible for 17.9 million deaths per year, 85% of which are due to heart attack and stroke. The WHO stated that most CVD can be prevented by adopting a healthy lifestyle, e.g., reducing the use of alcohol and tobacco as well as improving diet and physical activities. Consequently, detection and management using counseling and medicines, as appropriate, is a promising strategy to reduce CVD risk factors.

**Figure 2:**
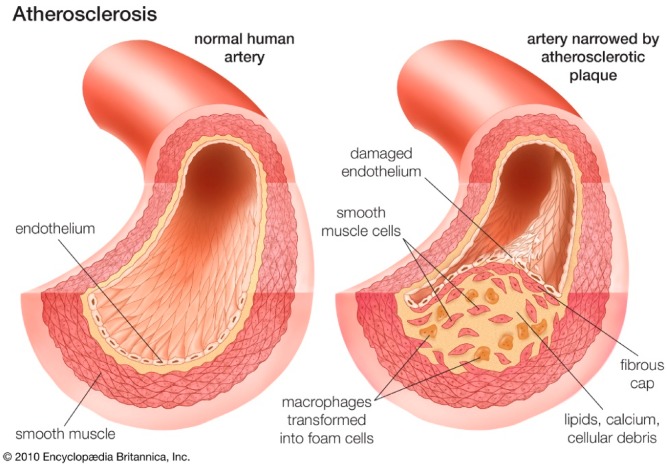
Atherosclerotic plaque formation in a damaged endothelium.

The dominant pathogenesis of CVD is represented by ATS, which is an inflammatory disease that is increasing worldwide as a result of the adoption of the Western lifestyle, and it is likely to reach epidemic proportions in the coming decades [7]. The major direct cause of CVD appears to be the atherosclerotic plaques [8]. Nowadays, it is well-known that ATS is a chronic metabolic and inflammatory process affecting the intima of medium-sized and large arteries. This process is characterized by the formation of plaques made of a cholesterol-rich core (atheroma) surrounded by a fibrous cap (Figure 2). ATS risk factors such as smoking, hypertension, dyslipidemia, diabetes, a sedentary lifestyle, and obesity lead to the activation (dysfunction) of the endothelium [9]. The activated endothelium exhibits an increased permeability, generates reactive oxygen species (ROS), and expresses inflammatory adhesion proteins and chemokines, contributing to the formation of the atherosclerotic plaque, which can be classified into types I and II (early lesions) or types II to VI (advanced lesions) on the basis of the lesion progression [9]. In addition, neoangiogenesis contributes to the progression of atherosclerotic plaque and complications [10].

## 3. Inflammation

**Figure 3:**
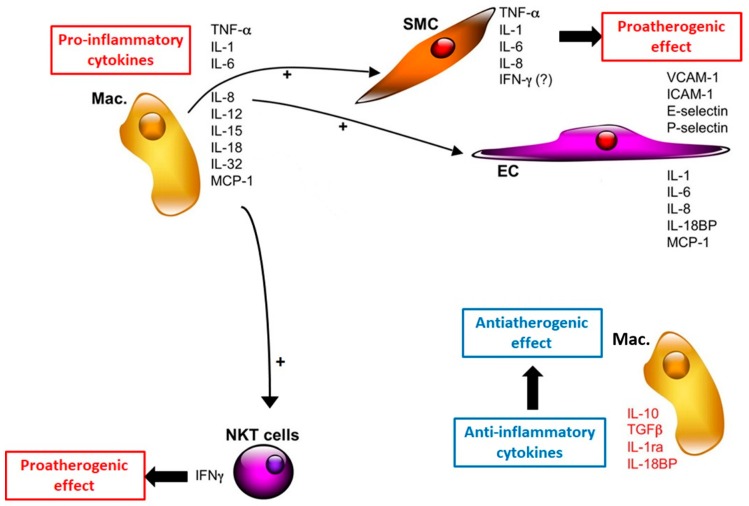
Cytokines involved in atherogenesis (adapted from [11]); Mac. = macrophage, SMC = smooth muscle cells.

Cytokines are often classified in pro-inflammatory (tumor necrosis factor-α (TNF-α), interleukin-1 (IL-1), interleukin-12 (IL-12), interleukin-18 (IL-18), interferon γ (IFNγ)) or anti-inflammatory (interleukin-4 (IL-4), interleukin-10 (IL-10), interleukin-13 (IL-13), transforming growth factor-β (TGF-β)) molecules, according to their activities during the inflammation process (Figure 3). Cytokines, secondary mediators of inflammation, are produced by monocytes, neutrophils and natural killer T (NKT) cells in response to microbial infection, toxic reagents, trauma, antibodies, or immune complexes. After inflammation has been triggered, there is a release of cytokines, the production of which is maintained and amplified by several other factors [11].

Caspases are cysteine proteases that have an important role in the execution of apoptosis. A subfamily of caspases known as inflammatory caspases is involved in innate immunity. Caspase-1 is the prototypic member of this subfamily: its activation requires the assembly of the inflammasome, which is a unique intracellular complex that cleaves and activates IL-1 and IL-18 and contributes to the production of all the other cytokines [11]. Recently, the activation of Nucleotide-binding domain and Leucine-rich repeat Receptor containing a Pyrin domain 3 (NLRP3) inflammasome activation, the oxidative stress causing immune cell dysregulation, and chronic infections have showed a pivotal role in ATS and in inflammaging, which is a condition involved in CVD [12,13].

## 4. Role of Oxidative Stress

Oxidative stress results from an imbalance between free radicals and antioxidants in the body that could promote endothelial dysfunction and lead to cardiovascular dysfunctions [14].

There are tight relations between ROS generation and vascular functions in the normal physiological state and various pathologies, ATS being among them [15]. A high concentration of ROS can damage endothelium cellular structures and components, resulting in cellular death [16]. Cells expressed in the atherosclerotic plaque can generate ROS in response to activation by cytokines (TNF-α, IL-1), growth factors (platelet-derived growth factor (PDGF)), vasoactive peptides (angiotensin II), and platelet-derived products (thrombin, serotonin). Although different enzymes are present in the atherosclerotic plaque, NADPH oxidase-like activity appears to be the most important enzymatic source of ROS in the vascular wall [11]. 

### Model for the Study of Endothelial Dysfunction 

Endothelial cells (EC) lining the blood vessels are very sensitive to injury caused by oxidative stress [17]. The injury leads to compensatory responses that alter the normal homeostatic properties of the EC, increases the adhesiveness of the endothelium to leukocytes and platelets, as well as its permeability [18], and induces a procoagulant state and the release of vasoactive molecules, cytokines, and growth factors. If the inflammatory response is not effectively neutralized or the offending agents are not removed, the process can continue indefinitely [18]. 

Human Umbilical Vein Endothelial Cells (HUVEC) have been considered a good standard model for EC in normal and diseased conditions [19,20,21,22,23,24]. HUVEC were cultured for the first time in 1973 and isolated by the perfusion of healthy donors’ umbilical veins with trypsin or collagenase [20]. HUVEC offer several advantages not only because they are relatively easy to recover and isolate from the umbilical vein, but also because they can be made to proliferate, and they can be maintained by a standard protocol. Moreover, HUVEC have been shown to be responsive to physiological and/or pathological stimuli such as high glucose, lipopolysaccharide (LPS), and shear stress [21,22,23].

Many in vitro studies performed on EC demonstrated the beneficial effects of natural products and their derivatives in protection from aging and oxidative stress [25,26,27].

## 5. Nutraceutical Intervention

The term “nutraceutical” derives from the fusion of the words “nutrition” and “pharmaceutical” [28]. According to DeFelice, nutraceutical can be defined as “a food (or a part of food) that provides medical or health benefits, including the prevention and/or treatment of disease”.

Since the term nutraceutical has no regulatory meaning in marketing, different definitions have been proposed to help distinguish between functional food, nutraceuticals, and dietary supplements [29,30]. In 1994, Zeisel [31] provided two additional useful definitions of nutraceutical and functional food. A nutraceutical can be defined as “a diet supplement that delivers a concentrated form of a biologically active component of food in a non-food matrix to enhance health”. Functional food is not a dietary supplement, but it includes “any food or food ingredient that may provide a health benefit beyond the traditional nutrients it contains” [32].

For these reasons, the interest in nutraceuticals and functional food has gained ground for its safety and potential nutritional and therapeutic effects. From here, it can be stated that because any functional food/nutraceutical is a source of macro and micronutrients, depending on the dose, it has the potentiality to be used as a drug [33].

In particular, nutraceuticals have showed a physiological benefit or provided protection against chronic inflammatory disorders, such as CVD [34]. 

Therefore, lowering inflammation is the most promising strategy for the prevention of atherosclerosis and its complications. Many clinical studies, e.g., the Lyon Diet Hearth Study [1], have demonstrated the protective effects in the primary and secondary prevention of CVD [2,3]. The consumption of plant derivatives, with a high intake of fruit and vegetables, such as plant sterols/stanols, red year rice, green tea catechins, curcumin, berberine, garlic etc., reducing physiological threats, including CVD and ATS risk factors [35], and improving the immune responses and defense system [36,37,38], could be used in monotherapy or combination therapy to significantly reduce CVD-related complications [39].

The constant increase of the nutraceutical market led the nutraceutical industry to develop innovative research in the delivery systems of molecules that have poor solubility or adsorption. These molecules without an appropriate oral formulation have limited efficacy [40].

## 6. Polyphenols and Sweet Cherry (*Prunus avium* L.)

**Figure 4:**
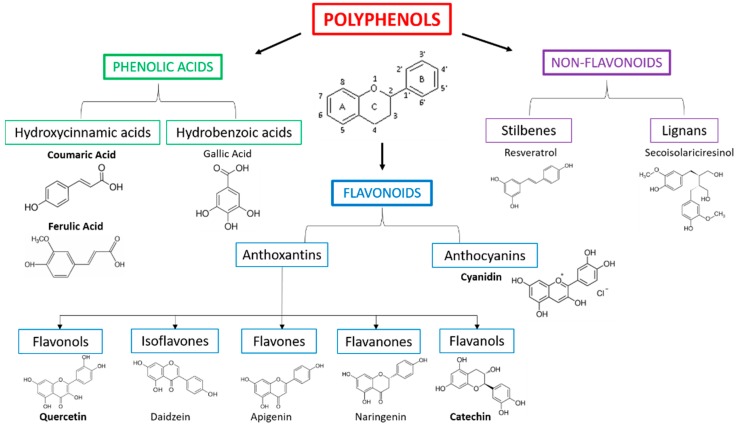
Schematic classification of polyphenols and examples of chemical structures. The main molecules present in sweet cherries are represented in bold.

Polyphenols are biologically active substances that are contained in plants derivatives or produced as secondary metabolites, which can be chemically distinguished into three main classes: phenolic acids, flavonoids, and non-flavonoids (stilbenes—resveratrol and lignans) (Figure 4). Polyphenols found in fruit, vegetables, nuts, and their derivatives have antioxidant and anti-inflammatory activities. Among phenolic acids, hydroxicynnamic, e.g., p-coumaric acid, and hydrobenzoic acids, e.g., gallic acid, have important antioxidant properties. Most polyphenols are represented by flavonoids, such as anthocyanins (cyanidin) and anthoxantins (flavonols—quercetin, flavanols—catechin etc.), which have both antioxidant and anti-inflammatory properties [41,42,43]. Flavonoids are found in chocolate, tea, and wine. Since oxidative stress is a determining factor in many chronic and degenerative pathologies, e.g., ATS, numerous efforts have been made to study antioxidant compounds that could prevent these diseases and hamper their progression. Indeed, numerous types of polyphenols (e.g., p-coumaric acid, gallic acid, and ferulic acid) have been found to have radical scavenging and antioxidant activity [44]. The literature also shows by in vitro and/or in vivo models that polyphenols could reduce the inflammation, inhibit the edema, and stop the progression of tumors, as a virtue of their proapoptotic and anti-angiogenic actions. In addition, they could modulate the immune system, prevent the bones disturbances associated with the osteoporosis, increase the capillary resistance by acting on the constituents of blood vessels, protect the cardiovascular system, etc. [45].

Among plant products, cherry fruit has been studied for its nutritional properties and beneficial effects [46,47]. Cherries are within the Rosaceae family and belong to the genes *Prunus* and subspecies *Cerasus*, according to the Linneus classification. Sweet cherry (*Prunus avium*) and tart or sour cherry (*Prunus cerasus*) have global trading importance and are now growing widely around the world. Depending on pre- and post-harvest factors, sweet cherry contains high levels of nutrients and bioactive compounds, which present various health benefits [48,49,50]. Średnicka-Tober et al. [51] showed the in vitro antioxidant potential of different cultivars of commercial sweet cherries, having a high variability in phenolics profile, and the ability to prevent disease. Other studies [52,53] confirmed the high phenol content variability and demonstrated that local sweet cherry varieties represent an interesting source of bioactive molecules and promote sustainability and biodiversity. Several clinical studies have showed that cherry fruit or juice consumption plays an important role in inflammatory diseases [54,55,56] by preventing or reducing inflammation related to muscle damage from intense strength exercise and also by accelerating recovery from strenuous physical activity. Ben Lagha et al. [57] reported that the tart cherry fractions and their bioactive constituents have antiplaque action due to their ability to inhibit the adherence properties of oral pathogens and increase the epithelial barrier function. Moreover, a recent study has confirmed the importance of cherry fruit in ATS risk factors prevention due to the effect of its polyphenols to reduce inflammation and endothelial dysfunction [58]. Nowadays, the interest is also moving toward the possibility of using coffee cherry extracts for brain health improvement, although further studies are required [59].

The most representative molecules in cherries are polyphenols, such as phenolic acids and flavonoids (see Figure 4), which also represent the most abundant antioxidants in the diet [60]. In particular, cherry extracts (CE) have a high content in phenols that reflects their nutraceutical potential, which could prevent chronic diseases [52]. Anthocyanins, the water-soluble subclass of flavonoids, are the ones responsible for the red color of cherry fruit and for the major part of CE vasoprotective properties [61], e.g., anti-inflammatory, anti-atherogenic, and vasodilatory action in vitro [62]. The antioxidant ability and the protective effect against oxidative stress of CE phenols have been investigated and demonstrated mainly by in vivo studies [48]. Regarding their anti-inflammatory activity, some studies have demonstrated that anthocyanins, such as cyanidin-3-o-glucoside and quercetin, inhibit LPS-induced inflammation and the release of endothelial-derived vasoactive factors after vascular endothelial damage [43,63,64]. A possible CE phenols mechanism of action in the cells has been recently reported by Console et al. [65]. In particular, they demonstrated the activation of recombinant human mitochondrial carnitine/acylcarnitine transporter, which was reconstituted in liposomes, by polyphenolic extract from *Prunus avium* L, thus confirming their antioxidant properties and showing their involvement in the mitochondrial fatty acid oxidation pathway.

In our own experience, the sweet CE polyphenols from *Prunus avium* L. showed a potential antioxidant effect by protecting HUVEC against oxidative stress, in addition to an ability to reduce ROS [66]. CE also demonstrated the ability to reduce inflammatory cytokines production, which resulted to be as efficient as that of the strong anti-inflammatory drug dexamethasone [67]. 

However, the use of antioxidants extracted from fruit is restricted because of their poor oral bioavailability. Indeed, they have a poor intestinal absorption, because of the oxidation in the intestinal tract and metabolic degradation in liver. Hence, there is a low probability of finding effective concentrations of these substances in the blood that is their site of action for a long time after ingestion. From this, the importance is clear of a formulation that could maintain the structural integrity of polyphenols, increase their water solubility and bioavailability, and transport them toward the physiological target [45].

## 7. Nanotechnology in Nutraceutical

To avoid the problem of polyphenols’ low oral bioavailability, nanotechnology has been applied in nutraceutical and nanomedicine [68], which resulted in new drug delivery systems. The delivery of nutraceuticals provides protective mechanisms that are able to (1) maintain the active molecular form until the time of consumption and (2) deliver the active form to the physiological target within the organism [69].

From a technological point of view, nanocarriers are promising candidate as nutraceuticals delivery because they have a minimum influence on the appearance of final food products such as beverages [70].

Many types of nanosystems are increasingly studied to increase the stability of bioactives during storage and consumption, such as polymeric nanoparticles (NP), solid lipid NP, and liposomes. These nanosystems could deliver molecules with low bioavailability such as polyphenols [71]. In particular, nanoparticles are able to encapsulate phenolic compounds via hydrogen bonds and hydrophobic interactions, consequently increasing their aqueous solubility and preventing the oxidation in the GI tract [72]. NP having subcellular size improve the bioavailability of nutraceutical compounds. In particular, NP are able to prolong the polyphenols residence time in the GI tract, decreasing the intestinal clearance mechanisms and the interaction with the biological target [73]. Furthermore, NP can also penetrate into tissue through fine capillaries, cross the epithelial lining fenestration (e.g., in the liver), and are generally taken up efficiently by cells [74], thus allowing the efficient delivery of active compounds to target sites in the body.

Nanoparticles are solid colloidal particles with diameters in the range of 1–1000 nm. They are distinguished into nanospheres and nanocapsules. In particular, nanospheres have the drug dispersed inside the polymeric matrix or adsorbed on their surface. The polymeric matrix can be natural or synthetic: generally, natural polymers are preferred because of their biocompatibility, biodegradability, and relative non-toxicity; moreover, polymeric NP have various different structures and bio-imitative characteristics [75]. The ability of mucoadhesive polymeric nanoparticles to be internalized by cells and promote the absorption of phenolic compounds has been demonstrated [76,77]. In particular, more mucoadhesive NP were more able to enhance the bioavailability of the encapsulated drug than less mucoadhesive ones [78]. Among mucoadhesive polymeric matrices, natural chitosan and its derivatives are considered polymers of prime interest. Another polymer that has been approved by the United States Food and Drug Administration and European Medicine Agency and is considered one of the best biomaterials available for drug delivery [79] is synthetic poly(lactic-co-glycolic acid) (PLGA).

Therefore, bioavailability, targeting, and controlled release are the main advantages of using natural product-based nanomedicine [80]. The increased solubility and bioavailability, and improved sustained release by nanoencapsulation may elevate the phytochemicals’ bioactivities [81]. However, the problem related to the nanosystems potential toxicity needs to be investigated. The minimal systemic toxicity of a nanosystem, based on biodegradable and biocompatible PLGA, could be of some advantage and represent an alternative to chitosan derivatives [82].

From here, the idea emerged of developing nanosystems based on mucoadhesive chitosan derivatives, which showed the ability to promote polyphenols intestinal absorption and antioxidant activity for the entrapment and the delivery of CE polyphenols. In addition, a comparison was made between such nanosystems and those based on non-mucoadhesive PLGA, which have different physical–chemical properties, in order to evaluate and select the best delivery system for CE polyphenols [82]. 

In addition to polymeric nanocarriers, an interesting strategy for drug delivery is represented by lipid-based nanocarriers, including vesicles, which were introduced as drug delivery vehicles for the first time in the 1970s. Vesicles are denominated either as liposomes, if the amphiphilic molecules are represented by phospholipids, or niosomes if they are based on non-ionic surfactants [83,84]. Liposomes have a spherical bilayer structure with sizes ranging from 20 nm to several μm. They are made of natural or synthetic phospholipids and cholesterol, and they can be loaded with either hydrophilic or hydrophobic molecules. Liposomes have shown many advantages, such as cell-like membrane structure [85,86], high biocompatibility, low immunogenicity, protection of the drugs or active groups, prolongation of drug half-life, reducing drug toxicity, and increasing efficiency. Moreover, structural and surface modifications can be made by using targeting ligands to generate a novel generation of liposomes and promote receptor-mediated endocytosis [87], thus expanding the application of liposomes in biomedicine [88]. Liposomes can be classified on the basis of their structural parameters, preparation methods [89], composition, and therapeutic applications. Their ability to encapsulate natural substances, e.g., plant-derived essential oils, grape seed extracts (GSE), curcumin, and enhance their antioxidant and anti-inflammatory activity has been demonstrated [90,91,92]. The liposome coating with chitosan led to a system for the controlled and sustained release of GSE polyphenols in water-based food [92]. Then, liposomes represent innovative vectors for the prolonged and sustained release of nutraceuticals and other active molecules, and their structure can be easily modified for multiple specific therapeutic applications.

A more recent trend in nanotechnology is represented by the use of complex systems. However, there are only a few data regarding the application of these systems as vehicles for nutraceuticals. Ma et al. [93] demonstrated the ability of nitric oxide-releasing chitosan nanoparticles (GSNO-Ch NP) to maintain the quality of sweet cherries during cold storage, thus improving their antioxidant properties. In effect, the authors showed that the combined treatment with S-nitrosoglutathione (GSNO) and Ch NP can preserve the soluble solid content and enhance the activity of antioxidants enzymes, in addition to reducing nitric oxide production, during its storage, better than GSNO or Ch alone.

### 7.1. Nanoparticles Based on Chitosan Derivatives 

**Figure 5:**
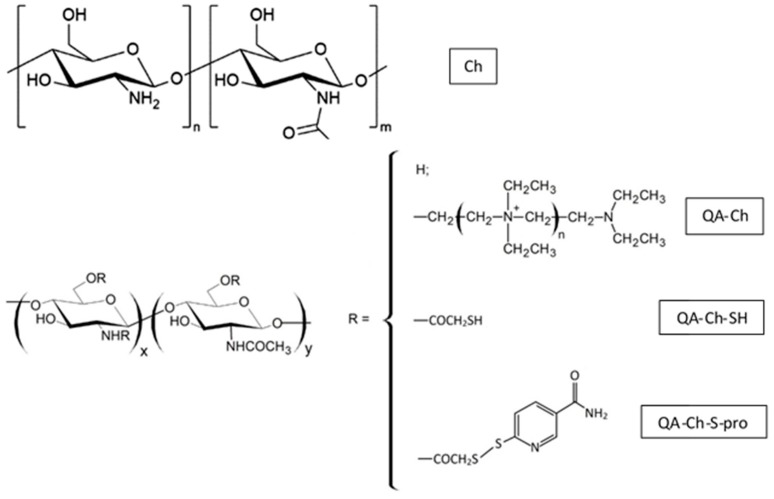
Chemical structures of chitosan (Ch) derivatives: quaternary ammonium chitosan (QA-Ch), its thiolated derivative (QA-Ch-SH) and S-protected quaternary ammonium chitosan (QA-Ch-S-pro).

Chitosan (Figure 5) is a cationic polysaccharide composed of d-glucosamine and *N*-acetyl-d-glucosamine units, which are linked by β-(1,4)-glycosidic bonds. It is obtained by the incomplete deacetylation of chitin, which is a homopolymer of β-(1,4)-linked *N*-acetil-d-glucosamine present in the shell of crustaceans and molluscs, the cell walls of fungi, and the cuticle of insects. Chitosan is biocompatible, biodegradable, mucoadhesive, and non-toxic, and it has antimicrobial, antiviral, and immunoadjuvant activities.

Chitosan obtained by a heterogeneous reaction is not soluble in water, although it is soluble in acid conditions. Water-soluble chitosan is instead obtained with homogeneous reaction. The acetylation of highly deacetylated chitin can also produce soluble chitosan. As a result, chitosan is available on the market in various forms that are different in molecular weight (MW) and deacetylation degree. Moreover, chitosan can be chemically modified because of the presence of –NH_2_ and –OH groups on the repetition units, leading to different derivatives. Chitosan has been found to enhance drug penetration across the cell monolayer, such as the intestinal epithelia [94]. Due to its absorption-enhancing effect, chitosan can be used for the development of new therapeutic drug delivery systems [95] administered by the oral route. Thus, the mucoadhesive properties of chitosan could be applied in nanomedicine with the purpose of improving the effectiveness of nutraceuticals and drug delivery systems in age-related and diet-related diseases, e.g., ATS [96].

However, the use of chitosan is restricted because of its limited mucoadhesive strength and low water solubility at neutral and basic pH. For these reasons, various chemical modifications of chitosan have been studied in order to improve its solubility and consequently its applications [97]. In its protonated form, chitosan facilitates the paracellular transport of hydrophilic drugs combining the bioadhesion to a transient widening of the tight junction in the membrane. However, it is incapable of enhancing the absorption in the more basic environment of the small intestine. Therefore, positive charges have been introduced on the chitosan polymer chains [98,99] to obtain chitosan derivatives with increased solubility properties, especially at neutral and basic pH values.

A promising class of chitosan derivatives called N,O-[N,N-diethylaminomethyl(diethyldimethyleneammonium)_n_methyl chitosan, or quaternary ammonium chitosan (QA-Ch) (Figure 5), was prepared by reacting chitosan with 2-diethylaminoethyl chloride under different conditions [4].

QA-Ch has a high fraction of free, unsubstituted, primary amino groups that are potentially available for the covalent attachment of thiol-bearing compounds via the formation of 3-marcaptopropionamide moieties. This has led to water-soluble thiolated chitosan-quaternary ammonium conjugates (QA-Ch-SH), which are also called thiomers (Figure 5). Thiol groups tend to keep the polymer adherent to the epithelium by reacting with the thiol groups of the epithelium mucus to form disulfide bonds, thus favoring the permeability-enhancing action of the positive ions. The synergism of quaternary ammonium and thiol groups has been evidenced [100]. Indeed, it has been demonstrated that the thiomer was more effective than the non-thiolated parent polymer in promoting absorption. The quaternary ammonium ions of the thiomer are responsible for the permeabilization of epithelium and the polymer mucoadhesion, while the thiols increase the latter. This synergistic effect is the basis of the polymer bioactivity [100]. 

To confirm the NP penetration mechanism, sections of the intestinal wall were observed under a fluorescence microscope following incubation with NP [101]. Microphotographs showed discrete fluorescent spots across the gut section, which were representative of integral NP penetration from the mucosal to serosal side of the intestine. This demonstrated that the NP did not disintegrate in their transit across the intestinal wall [101].

Despite the innumerable qualities, the thiomers have shown instability problems in solution; in particular, the thiol groups can be subject to oxidation at pH values ≥ 5. The early oxidation of thiols can limit the interaction with glycoproteins in the mucus, drastically reducing the effectiveness of these polymers. To overcome this problem, it was necessary to design and develop a second generation of oxidation-stable thiomer, called S-protected chitosan (QA-Ch-S-pro) (Figure 5). The protection of the sulfhydryl ends with mercaptonicotinamide groups allows increasing the mucoadhesive and cohesive properties of the thiomers, independently of the pH of the environment. Moreover, the amplified adhesive properties of the polymer make it possible to prolong the contact time with the mucosal membranes, the residence time of any vehiculated drugs, or small molecules, thus increasing the concentration gradient of these at the absorption site. Consequently, the more facilitated transport allows increasing the bioavailability of the drugs, with consequent reduction of the dose and the frequency of administration. Thus, chitosan-S-protected polymers can be considered a promising category of mucoadhesive polymers for the future development of new, effective, and safe non-invasive delivery systems for polyphenols.

**Figure 6:**
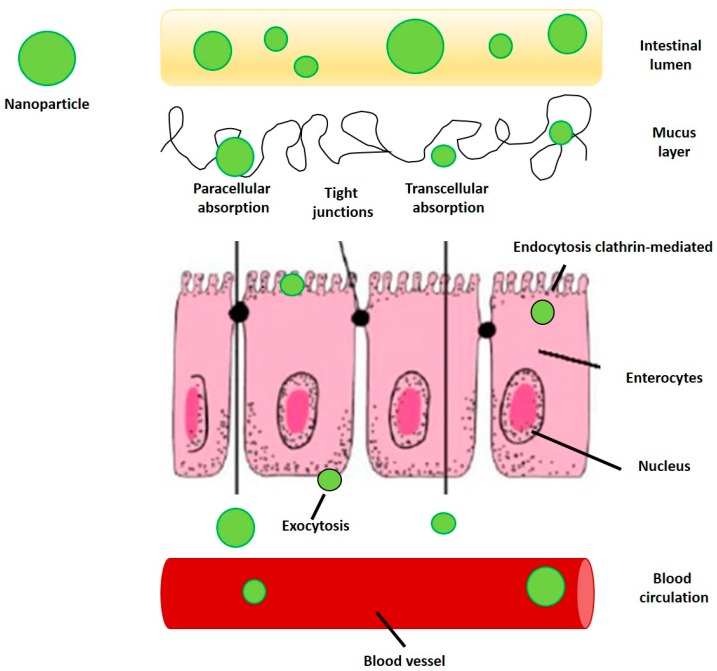
Cellular uptake of nanoparticles (NP) carrying polyphenols by intestinal epithelial cells.

The antioxidant, anti-inflammatory, antidiabetic, and anticancer properties of chitosan and its derivatives [96], especially when combined with such natural antioxidants as polyphenols, are promising for the prevention, delay, mitigation, and treatment of age-related dysfunctions and diseases, such as CVD. Moreover, NP are able to enhance the absorption of phenolic compounds because they are able to disrupt the tight junctions of biological membranes and can be directly uptaken by epithelial cells via endocytosis (Figure 6) [102].

To prepare Ch-der NP, different techniques have been used [103], but the choice of a particular method should consider the nature of the drug to be entrapped, the delivery system, the administration route, and the absorption site. One of the established methods for the preparation of mucoadhesive Ch-der NP, which is intended for oral absorption, is the ionotropic gelation with de-polymerized hyaluronic acid (HA) [104], which is very simple because it does not require the use of organic solvents. The NP are obtained by the addition of a solution of HA containing or not the drug to a dilute solution of chitosan, under stirring. Nanoparticle size strictly depends on the concentration of both chitosan and HA. The efficacy of Ch-der NP prepared with this method to encapsulate red grape polyphenols, thereby promoting their oral absorption and producing beneficial effects on endothelial cells, has been demonstrated [76,77]. Moreover, a recent study on Caco-2 cells demonstrated that Ch-der NP were easily internalized by adsorptive endocytosis [97].

Chitosan and its derivatives were used also to prepare nanoparticles complex systems. Ba et al. [105] prepared zein-carboxymethyl chitosan-tea polyphenols (zein-CMCS-TP) for the delivery of β-carotene. These ternary complexes had more stability against heat and acid conditions and antioxidant activity than single protein and protein-polysaccharide binary systems. Zein NP coated with alginate/chitosan were used also to encapsulate resveratrol [106]. These complexes reduce the photodegradation of resveratrol, could improve its stability, and could represent a useful potential delivery system for application in functional food and pharmaceutical products.

### 7.2. Poly(Lactic-co-glycolic Acid) Nanoparticles

**Figure 7:**
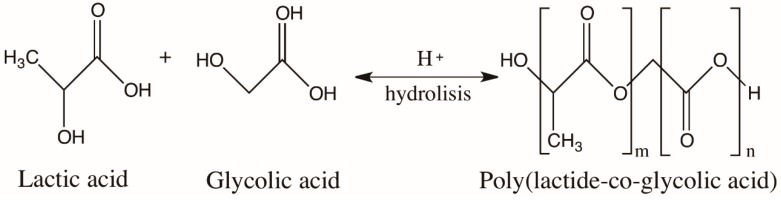
Degradation of poly(lactic-co-glycolic acid) (PLGA) based on the hydrolysis of the copolymer.

The polyester PLGA is a synthetic copolymer of poly lactic acid (PLA) and poly glycolic acid (PGA) (Figure 7). PLGA is biocompatible and biodegradable, and it is used not only as a delivery vehicle for drugs, proteins, and other macromolecules, but also for the development of NP containing nutraceuticals [107]. It is soluble in a wide range of common solvents including chlorinated solvents, tetrahydrofuran, acetone, or ethyl acetate. In water, PLGA is degraded by the hydrolysis of its ester linkages (Figure 7). PLGA NP can be used to encapsulate either hydrophilic or hydrophobic small molecules by using different formulation methods.

The most common technique for the preparation of PLGA NP that is able to encapsulate small hydrophilic molecules is the double emulsion technique (w/o/w), which is a modification of the emulsification-solvent evaporation technique [108]. PLGA NP are internalized by cells partly through pinocytosis and also through clathrin-mediated endocytosis and enter the cytoplasm within 10 min of incubation [107]. The controlled release, biocompatibility, and biodegradability properties of PLGA NP have produced an overall decrease in cytotoxicity; therefore, they have been used as delivery systems for polyphenols rich-materials from fruit and other nutraceuticals [108,109,110,111]. The negative surface charges of PLGA could also be modified by PEGylation of the polymer [112] or coating NP with chitosan [113]. In the first case, NP with a neutral surface were obtained; in the second case, the NP surface was positively charged. In both cases, the NP cellular uptake was improved. Another advantage of using PLGA nanoparticles or nanospheres is in the possibility of reducing local inflammation through a long-term treatment, thanks to the slow biodegradation of NP and the consequent release of the drug [114]. In particular, PLGA NP have been successfully used for the preparation of polyphenol nanoformulations in cancer therapy [115].

In addition to simple PLGA NP, more complex and recent strategies have been applied for the delivery of nutraceuticals different from cherry. PEG-lipid-PLGA hybrid NP were prepared by Yu et al. [116] to enhance the liposolubility and the oral delivery of berberine, which is a natural compound that presents potential anti-cancer and anti-inflammatory activity. Complex nanoparticles systems could be also prepared by the combination of PLGA with Ch. Abd-Rabou et al. [117] used polyethylene glycol/chitosan-blended PLGA (PLGA-Ch-PEG) to prepare *Moringa oleifera* leaves extract-loaded nanocomposites, which could be used as a natural source of anti-cancer compounds. Another study reported the possibility of co-encapsulating Nigella sativa oil (NSO) and plasmid DNA (pDNA) in chitosan-PLGA NP, in order to improve the gene therapy for Alzheimer neurodegenerative disease [118].

### 7.3. Liposomes

**Figure 8:**
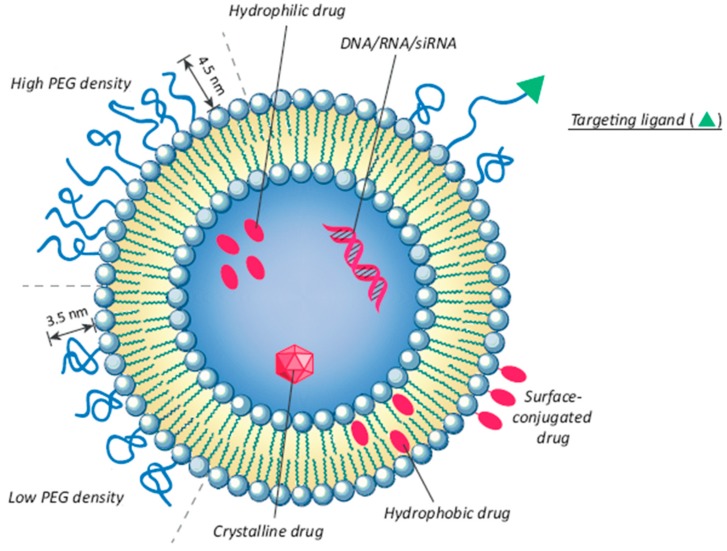
Structural and design considerations for liposomal drug delivery (adapted from [87]).

Liposomes are bilayer vesicles with an aqueous core entirely covered by a phospholipid membrane. They are attractive encapsulation systems for water-soluble phenolic compounds [119] (Figure 8). 

The thin layer evaporation technique is one of the simplest and most used methods [120] to prepare liposomes by hydrating lipid films [85,121,122], which involves the encapsulation of active principles in the organic phase (with lipophilic actives) or in the aqueous phase (with hydrophilic ones) during the initial steps of liposomal preparation. However, using this technique, the encapsulation efficiency is generally higher with lipophilic molecules than with hydrophilic ones. Another limitation of using conventional liposomes is represented by a rapid elimination from the bloodstream, which could reduce the therapeutic efficacy [123].

In the food area, these vesicles could be used for the encapsulation of functional bioactives. Among the bioactive substances, the essential oils have been thoroughly studied, since many of them have strong antioxidant and antimicrobial properties [124]. However, the difficulties with their dispersion in aqueous formulations and their high oxidation sensitivity require their encapsulation in water-dispersible systems and protection from degradation. A recent work [125] demonstrated the ability of multilamellar liposomes prepared by the dry film hydration technique to incorporate essential oil from Brazilian cherry (*Eugenia uniflora* L.) leaves, which is a plant that is known for its anti-inflammatory properties.

Liposomal aqueous dispersions have low stability; therefore, anhydrous liposomal preparations have been studied. Anhydrous preparations have the advantage of being stable and can be hydrated to regenerate the liposomal dispersion at the time of use. For this reason, transforming the aqueous liposomal dispersion into powder means creating a release system that is more fit for industrial production. This was the goal of Akgün et al. [126], who showed a promising industrially applicable delivery system for sour cherry phenols that were efficiently loaded in a liposomal powder incorporated into a stirred-type yoghurt system. Since the spray-drying process did not degrade phenolic compounds encapsulated in liposomes, this technique could represent another strategy for reducing polyphenols degradation and enhancing their beneficial activity.

## 8. Intestinal Absorption

The oral route is the preferred one for drug administration because it is the physiological mechanism of nutrients and other exogenous molecules [127]. A drug administered by the oral route is mainly absorbed in the small intestine. The small intestinal epithelium is mainly composed of enterocytes, which have well-ordered projections, called microvilli, on their apical side. Microvilli increase the absorptive area, making up a total intestinal surface area of 300–400 m^2^. The intestine also comprises mucus-secreting goblet cells, which are the second most abundant cell type.

Mucus has an essential role in the GI tract. In fact, it has transport activity as well as lubricant and protective properties. It is the first physical barrier encountered by biopharmaceuticals after their oral administration [128]. Mucus is a complex hydrogel composed of proteins, carbohydrates, lipids, salts, antibodies, bacteria, and cellular debris. The main protein components of mucus are mucins, which are responsible for the gel properties of mucus [129]. An example of the dynamic barrier properties of mucus is represented by its ability to act as a selective barrier to the diffusion of acids, due to interactions that change depending on the environmental pH and pKa of the acid.

Since the primary site of absorption after oral administration is represented by the small intestine, rather than the colon [127], it is important to establish the best epithelial cells-based model that is able to simulate the intestinal barrier, in order to evaluate the nutrients intake. After being transported across the epithelial lining, molecules reach the lamina propria, which contains a network of capillaries responsible for their drainage into blood circulation and thus to their action site. All epithelial cells are interconnected by tight junctions, which have an important role in retaining the polarization of the cells and maintaining the integrity of the epithelium [130].

Nanoparticles have the potential to enhance the absorption of phenolic phytochemicals because they are able to disrupt tight junctions and/or they could be directly uptaken by epithelial cells via endocytosis [102] (see Figure 6).

The in vitro model most widely accepted to study the human oral drug absorption is the colon epithelial cancer cells (Caco-2) monolayer. Caco-2 clones from adenocarcinoma have morphologic and functional characteristics similar to enterocytes: e.g., they show tight junctions, apical and basolateral sides, and a brush border with microvilli on the apical surface. However, these Caco-2 monolayers have several limitations. One of these is represented by tight junctions being tighter than those present in the small intestine. In addition, they are more similar to colon epithelium cells, as they have a reduced permeability to drugs through the paracellular route. Hence, many research groups have proposed to use the co-culture of Caco-2/methotrexate mucus-secreting subclones HT29-MTX, as a model that is able to mime the human intestinal epithelium better than the simple Caco-2 monolayer. The mucus-producing HT29-MTX cell line is used as a model to study the mucus role in the transport of drugs through the intestinal tract. Mucus-secreting goblet cells are usually obtained from adenocarcinoma cell line HT29. HT29 cells are treated with methotrexate to get mature goblet cells, which are so-called HT29-MTX.

### Triple Cell Co-Culture (Caco-2/HT29-MTX/Raji B) as a Model of Study

**Figure 9:**
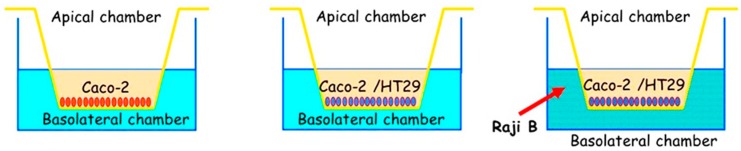
Scheme of Caco-2/HT29 (HT29-MTX)/Raji B triple cell co-culture model preparation (adapted from [127]).

A more recent in vitro model based on a triple cell co-culture of Caco-2/HT29-MTX/Raji B, as represented in Figure 9, has been developed in order to reproduce the intestinal epithelium [127,130]. Caco-2 cells cultured with Raji B lymphocytes acquire the M cell phenotype. Caco-2 cells losing the brush border organization, the microvilli, and the typical digestive function from enterocytes play an important role in the immune system, and they have the ability to take up bacteria, viruses, nanoparticles, and microparticles by endocytosis. Previous studies [127,130] proved that the three cell types, when cultured together, present the features of the human intestinal barrier.

In our studies [66,67,82], we tested Ch-der and PLGA NP on both HUVEC and Caco-2 cells in order to evaluate NP cytotoxicity, their ability to protect polyphenols from degradation in the GI, and the mucoadhesive properties that are able to promote intestinal absorption. Our results demonstrated that Ch-der NP, based on mucoadhesive QA-Ch and QA-Ch-S-pro derivatives, were able to encapsulate CE polyphenols and protect them from GI degradation [66]. In particular, QA-Ch and QA-Ch-S-pro NP enhanced the anti-inflammatory and antioxidant activity, respectively, of the lowest CE polyphenolic concentration tested (2 µg/mL). This was ineffective when non-encapsulated [66,67]. PLGA NP were able to encapsulate higher polyphenolic concentrations, maintain their beneficial activities, and promote intestinal permeation [82]. Both Ch-der NP had the ability to reduce ROS production, but only QA-Ch-S-pro NP significantly protected HUVEC from oxidative stress [66], which was probably because of the highest affinity between CE and NP. It is probable that the presence of protected thiol groups on the surface, acting as reducing groups [131], enhances the polyphenols’ antioxidant effect. Moreover, QA-Ch-S-pro NP were able to promote CE polyphenols intestinal permeability through the in vitro triple co-culture model based on epithelial cells (Caco-2/HT29-MTX/Raji B) better than non-mucoadhesive PLGA NP [82]. For its part, QA-Ch NP showed the ability of reducing inflammatory cytokines production, nitric oxide, and NLRP3 production in stressed HUVEC, to the same extent as the anti-inflammatory synthetic drug dexamethasone [67]. Although all the NP types were efficiently internalized by HUVEC after 2 h of incubation, the mucoadhesive properties and the positive surface charge of Ch-der NP showed higher cellular interaction than the non-mucoadhesive and negatively charged PLGA NP [67].

The results obtained have shown that all the types of NP tested are promising from the nutraceutical standpoint. Chitosan NP, thanks to their chemical–physical properties, could be used as efficient transport systems for polyphenols; nevertheless, if higher polyphenolic concentrations are needed, the use of PLGA NP, as nanosystems with low cytotoxicity, could be more convenient [82].

Triple cell co-cultures of Caco-2/HT29-MTX/Raji B were also used as a model to assess the liposomes’ permeation ability. Otero et al. [132] demonstrated that non-encapsulated bacteriophages were able to cross the intestinal barrier with respect to the encapsulated ones, which was probably because liposomes containing bacteriophages had a prolonged residence time in the stomach, thus adhering to the intestinal wall and protecting phages until they release. In another study, Belubbi et al. [133] encapsulated nelfinavir mesylate (NFV) in liposomes and studied their permeability using the triple cell co-culture method. They found that the liposomes had a high NFV encapsulation efficiency, but no liposomes permeation was observed. However, the authors demonstrated that these liposomes were able to protect the drug in the gastric environment.

Although no liposomes containing polyphenolic compounds have already been investigated using triple cell co-cultures of a Caco-2/HT29-MTX/Raji B model, these results suggest that liposomes can protect the encapsulated drugs from degradation in the GI tract and that the triple cell co-culture model can yield sound information about polyphenols’ transcytosis.

## 9. Conclusions

Many clinical studies have reported that the consumption of cherries and their derivatives has a beneficial effect on human health. In addition, in vitro studies have demonstrated that natural polyphenols-rich sweet cherry extracts are able to protect endothelial cells from oxidative stress. Regarding inflammatory stress protection, CE was found to be as efficient as the most used anti-inflammatory synthetic drug dexamethasone.

The encapsulation of CE in nanoparticles based on chitosan derivatives improves the intestinal absorption of cherry polyphenols and enhances their antioxidant and anti-inflammatory activity. The mucoadhesive properties of the NP favor cellular internalization and promote the CE biological effects.

For all these reasons, the use of nanosystems based on chitosan derivatives represents a good and innovative strategy for the delivery of polyphenols from cherry extracts. PLGA-based nanosystems are a valid alternative in case higher polyphenol concentrations are needed. The differences in nutraceutical properties between the different types of nanoparticles loaded with cherry extracts have been attributed to the chemical differences between NP surfaces. Indeed, the surface properties of the nanoparticles influence their ability to be internalized by the cells and to cross the mucus that lines the intestine. 

Other types of carriers, such as liposomes, should be taken into account for the development of future delivery systems for polyphenols or essential oils. A more recent approach is the use of complex systems based on nanoparticles to enhance the stability of phytochemicals and thus preserve the therapeutic properties of the encapsulated bioactive compounds.

In conclusion, considering that the fresh cherry fruit is a seasonal fruit, the use of nanosystems protects CE from degradation in the GI, thus allowing cherry consumption and its benefits to not be limited by seasonality.
